# A Preliminary Evaluation of the Physiological Mechanisms of Action for Sleep Restriction Therapy

**DOI:** 10.1155/2013/726372

**Published:** 2013-11-20

**Authors:** Annie Vallières, Tijana Ceklic, Célyne H. Bastien, Colin A. Espie

**Affiliations:** ^1^École de psychologie, Université Laval, Québec, QC, Canada G1V A06; ^2^Centre d'étude des troubles du sommeil, Centre de recherche de l'institut universitaire en santé mentale de Québec, Québec, QC, Canada G1J 2G3; ^3^Centre de recherche du centre hospitalier universitaire en santé mentale du Québec, Québec, QC, Canada G1V 4G2; ^4^Nuffield Department of Clinical Neurosciences, Sleep & Circadian Neuroscience Institute, University of Oxford, Oxford OX3 9DU, UK

## Abstract

Our objective was to investigate the physiological mechanisms involved in the sleep restriction treatment of insomnia. A multiple baseline across subjects design was used. Sleep of five participants suffering from insomnia was assessed throughout the experimentation by sleep diaries and actigraphy. Ten nights of polysomnography were conducted over five occasions. The first two-night assessment served to screen for sleep disorders and to establish a baseline for dependent measures. Three assessments were undertaken across the treatment interval, with the fifth and last one coming at follow-up. Daily cortisol assays were obtained. Sleep restriction therapy was applied in-lab for the first two nights of treatment and was subsequently supervised weekly. Interrupted time series analyses were computed on sleep diary data and showed a significantly decreased wake time, increased sleep efficiency, and decreased total sleep time. Sleepiness at night seems positively related to sleep variables, polysomnography data suggest objective changes mainly for stage 2, and power spectral analysis shows a decrease in beta-1 and -2 powers for the second night of treatment. Cortisol levels seem to be lower during treatment. These preliminary results confirm part of the proposed physiological mechanisms and suggest that sleep restriction contributes to a rapid decrease in hyperarousal insomnia.

## 1. Introduction

Sleep restriction therapy for insomnia was developed by Spielman et al. in 1987 [1]. This behavioral intervention consists of restricting the time spent in bed to correspond to the estimated amount of time spent asleep by the patient [1]. Weekly changes are made to the time spent in bed as a function of the patient's clinical response. Since this first publication, sleep restriction therapy has been frequently included in cognitive-behavioral therapy for insomnia (CBT-I). Meta-analyses of nonpharmacological treatments of insomnia have shown that sleep restriction can be effective in decreasing sleep-onset latency and greatly increasing sleep efficiency in a relatively short time [2–4]. Another meta-analysis has shown that sleep restriction, applied with other behavioral treatment, benefits sleep of adults and older adults, with the exception of total sleep time [5]. Sleep restriction efficacy is well acknowledged by sleep clinicians and researchers [6, 7] who consider it an essential therapeutic component.

Sleep restriction mechanisms are seen as involving physiological and psychological processes of sleep. Despite the effectiveness attributed to sleep restriction, little is known about how or why it improves sleep. From a physiological point of view, it is suggested that the prescribed total time spent in bed entrains the biological clock and produces a mild sleep-deprived state that increases daytime wakefulness and thus the sleep homeostatic drive [1, 8, 9]. This in turn increases sleepiness at night that facilitates falling asleep, sleep consolidation, decreases rapid cortical activity, and increases slow wave sleep during early sleep cycles. In this sense, sleepiness at night and the cortical activity changes are markers of the physiological process of sleep restriction.

Sleepiness has been seen as an inevitable part of treatment that appears in the first weeks of treatment [10, 11] but is known to diminish by the follow-up assessment. Two recent studies evaluated sleep restriction alone and focus on sleepiness and vigilance [11, 12]. One evaluated daytime functioning of nine participants with chronic insomnia who received three sessions of sleep restriction [12]. This study demonstrated that sleep restriction immediately resulted in an impaired vigilance and an increased sleepiness at night. The second study [10] evaluated 16 participants with insomnia who received 4 weeks of sleep restriction and showed that sleep restriction therapy is associated with an elevated daytime sleepiness and impaired vigilance in the first three weeks of treatment. Although there is evidence of elevated sleepiness during the first week of sleep restriction, it is still uncertain how that could negatively affect treatment compliance as previously suggested [2, 4, 7] or how this sleepiness is related to cortical activity and to subjective sleep.

A few studies have investigated cortical activity with PSG and power spectral analysis (PSA) before and after CBT-I [9, 13, 14]. One study evaluated cortical activity in the presleep period before and after a combined sleep restriction, stimulus control, and relaxation treatment of insomnia [13]. Twelve participants with insomnia received 5 treatment sessions over 10 weeks of sleep restriction and stimulus control combined to relaxation and were compared to 14 normal sleepers. This study observed a decrease in the beta percent total power in the presleep period after treatment of insomnia, suggesting that these participants had, after treatment, less rapid cortical activity before going to bed. The second study investigated nine people suffering from chronic mixed-type insomnia [14]. Participants received eight weeks of CBT-I including sleep restriction therapy. Their PSG results showed a decrease in stage 2 sleep and an increase in both slow wave sleep (SWS) and REM sleep. The PSA showed a reduction in the beta activity during NREM sleep and an increase in SWS after CBT-I. Another study evaluated 16 participants with chronic insomnia and compared them to a placebo control group [9]. Participants in the treatment group received 8 weeks CBT-I including sleep restriction therapy. These authors found that CBT-I led to a greater rate of exponential decline in delta power over NREM sleep periods. No other frequency band changes were found to be significant after CBT-I. Therefore, there is some evidence that treatment of insomnia can produce a change in the rapid cortical activity of people with insomnia. However, because these studies used either a relaxation or a multicomponent treatment, it is not known whether these changes are due to sleep restriction therapy. Moreover, because PSG nights were done before and after treatment, they do not inform how and when sleep restriction works.

Cortisol is another possible physiological marker of sleep restriction, as it is a marker of the hypothalamus-pituitary-adrenal axis activity [15, 16]. Cortisol is associated with stress, cortical activity, and physiological hyperarousal. Therefore, according to the hyperarousal model of insomnia, cortisol should be higher in insomnia than in good sleepers. A few studies investigated the cortisol cycle in insomnia. People with insomnia were found to have a higher cortisol level in both the morning [15, 16] and evening [16] compared to good sleepers. Other studies have shown that relaxation may reduce overall cortisol level [17]. Therefore, it is likely that effective sleep restriction therapy that produces a decrease in total wake time could also lead to a decrease in cortisol levels. 

In summary, although sleep restriction is currently recommended as a treatment of insomnia and frequently included in CBT-I, very few studies have evaluated sleep restriction therapy mechanisms, other than the seminal work by Spielman and colleagues [1]. The present study tests a methodology that would be useful in evaluating sleep restriction physiological mechanisms in a larger program. A single-case design is used to explore the physiological mechanisms using continuous data collection throughout the treatment. The main goal of the present study is to explore the physiological mechanisms of sleep restriction: these mechanisms are explored using several measures of objective sleep, perceived sleepiness and alertness, and morning and evening cortisol levels. In addition, the study evaluates the efficacy of sleep restriction specifically, using data gathered daily in order to more closely follow the links between the treatment effects observed and the mechanisms under study. 

## 2. Methodology

### 2.1. Participants

Participants were recruited by physician referrals in the Greater Glasgow area. Inclusion criteria were as follows: (a) being between 18 and 65 years old; (b) presenting insomnia according to DSM-IV-TR [18] criteria; (c) reporting significant distress or daytime impairments (item 6 score of 2 or higher) as evaluated by the Insomnia Severity Index [19]; (d) cessation, at least one month prior to experimentation, of any sleep or other psychotropic medication that could alter sleep; (e) a baseline sleep efficiency lower than 75%; and (f) reporting a BDI score between “0” and “15.” Exclusion criteria were as follows: (a) presence of sleep state misperception insomnia defined as a marked discrepancy between subjective complaint and objective measure of total sleep time; (b) presence of another sleep disorder (apnoea and hypopnoea index > 15; periodic limb movement index > 15); (c) evidence that insomnia was related to a medical condition; (d) presence of major depression, anxiety disorder, alcohol/substance abuse, or any other psychopathology as diagnosed with the SCID-IV [20]; (e) being currently in psychotherapy; (f) regular use of medication interfering with sleep; and (g) use of antibiotics two weeks prior to the onset of the study or steroids within six months prior to study, which could affect cortisol level. Inclusion and exclusion criteria aimed at selecting severe insomnia in order to favor clear changes in the time in bed (TIB) from the beginning of treatment. The study was approved by the Research Ethics Committee of Greater Glasgow Health Board, at the Southern General Hospital (ethics reference number: 05/S0701/45).

Twenty-one eligible participants responded to the advertisement and underwent telephone screening. Sixteen were then excluded for the following reasons: sleep improved before the interview (*n* = 5); use of medication interfering with sleep or hypnotics (*n* = 6); the baseline sleep efficiency was higher than 75% (*n* = 4); and participants were no longer interested in the study (*n* = 1). Subsequent assessments included a semistructured sleep history interview [19] and a SCID-IV evaluation [20]. Thus, the final sample included five participants (1 male and 4 females) meeting DSM-IV-TR [18] criteria for primary insomnia. Only these five participants completed the experimentation from the beginning. Four participants completed the treatment and the whole experimentation while one completed the treatment but not the entire protocol. Their mean age was 41.1 years (ranging from 22 to 62) with an average education level of 15.2 years (ranging from 10 to 19). The average insomnia duration was 12.6 years (SD = 6.7). One participant presented sleep-onset insomnia only, and four presented mixed insomnia (sleep-onset, sleep maintenance, and/or terminal insomnia). Participants were free of any sleep medication for at least one month before entering the study.

### 2.2. Design and Procedures

#### 2.2.1. Design

**Figure 1:**
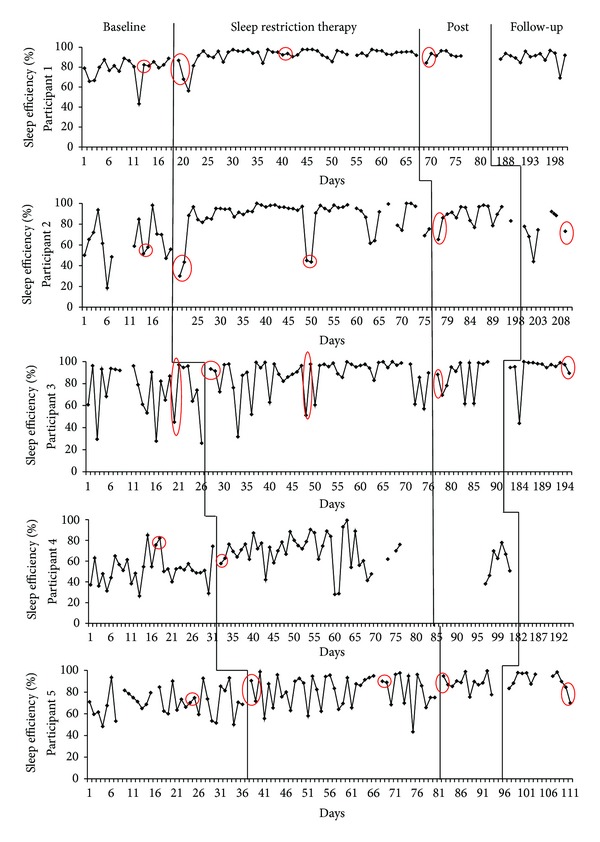
Daily sleep efficiency course for each participant. Circled data correspond to PSG nights.

**Figure 2:**
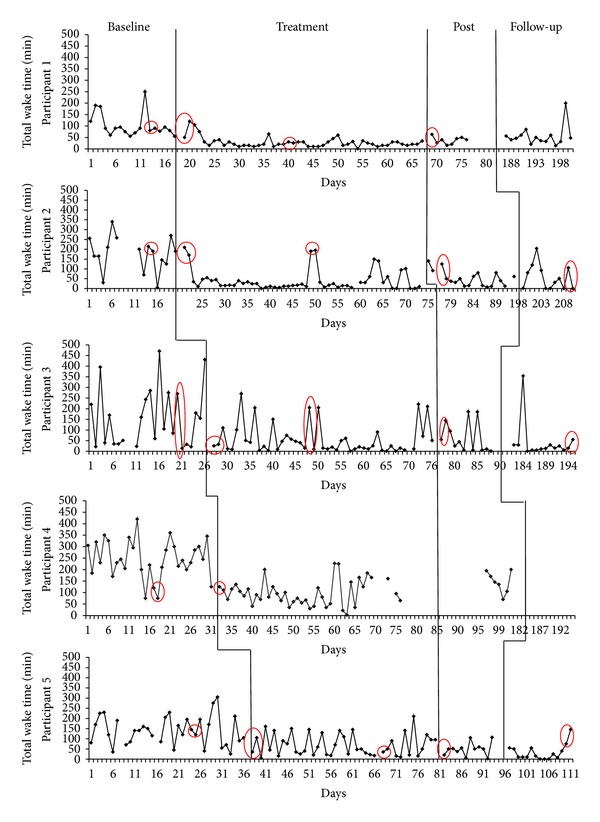
Daily total wake time course for each participant. Circled data correspond to PSG nights.

A single-case design called multiple baseline across subjects design [21] was used to evaluate the impact of sleep restriction on physiological variables. This particular single-case design provides a controlled investigation of treatment mechanisms [22]. Baseline length has to be different for each participant to ensure that the introduction of the experimental treatment (here, the sleep restriction therapy) occurs at a different time for each participant. This particularity of the present design provides a control for possible maturation. Maturation refers to natural changes over time of a participant's sleep that occur without treatment. Figures 1 and 2 illustrate the design by showing that each participant has a different baseline length.

#### 2.2.2. Procedure

After the screening procedure, participants began a baseline with varying lengths before treatment. At baseline and throughout the experimentation, participants completed continuous assessments of their sleep, cortisol, sleepiness, and alertness. They also wore an actigraph from baseline until the end of treatment. Participants completed the Insomnia Severity Index at three assessment periods: baseline, posttreatment, and 3-month follow-up. At five occasions, they also spent two consecutive weekday nights of in-lab PSG, with the first two being at the first baseline week and for which the very first night served as a screening night for other sleep disorders. The following three occasions of PSG nights were scheduled during the treatment interval: (a) two nights at the first sleep restriction therapy session, (b) two nights when sleep was considered stabilized, and (c) two nights after three weeks of sleep stabilization. The remaining two PSG nights were at a 3-month follow-up. 

Sleep was considered stabilized when sleep efficiency (SE) reached 85% or more, night-to-night variability was visually observed to have reduced relative to SE baseline, and a clinical judgment of progress was made. Night-to-night variability in sleep is an important feature of insomnia that is suggested to be an indicator of treatment responsiveness [23]. Moreover, the criteria used are closed to the clinical context in which the therapy takes place.

#### 2.2.3. Treatment

The sleep restriction administered in this study is outlined in a treatment manual [24] and follows reviewed recommendations [25]. The content and aim of each sleep restriction session are summarized in Table 1. The manual includes also answers to frequently asked questions (FAQ) in order to standardize answers given to participants and avoid delivering cognitive therapy or stimulus control therapy for insomnia (FAQ are available upon request from the first author). The first two nights of treatment were supervised and spent in laboratory as training for the sleep restriction procedures. Sleep restriction therapy consists of curtailing the time spent in bed to conform to the reported amount of time asleep. A sleep window is determined using the average of total sleep time reported by participants in their two baseline weeks of sleep diaries. The sleep window is increased by 15 minutes, contingent upon reaching a SE of 85% or more. When SE is between 80% and 85%, the sleep window is kept stable and when SE is lower than 80%, the sleep window is decreased to correspond to the total sleep time estimated. The lower limit of the sleep window is five hours. An educational component including basic facts about sleep is included in the treatment in order to give a more reliable treatment rationale to participants.

Sleep restriction therapy was introduced following each baseline period for four to six individual treatment sessions of 50 minutes. The first sessions are performed weekly until sleep is stabilized as previously described. Then, one more session is planned three weeks after sleep stabilization. Participants are instructed to increase their sleep window according to the same rules based on SE during weeks without a therapy session and at posttreatment after the supervised treatment periods. Also, they are invited to increase the sleep window by modifying their bedtimes to keep the lower limit constant throughout the treatment.

*Treatment Fidelity*. Several methodological strategies were used to monitor treatment fidelity. First, the multiple-baseline design controlled the beginning of the treatment, assuring that sleep restriction began only as previously determined. Second, the use of the manual facilitates treatment standardization and replication. Third, the two-night in-lab training of sleep restriction assured appropriate treatment application by participants. Finally, actigraph measures provided objective confirmation of treatment fidelity.

*Therapist*. A graduate student in psychology performed telephone screening. Treatment sessions and assessment interviews were led by a licensed clinical psychologist (AV) who had several years of experience in sleep restriction therapy.

### 2.3. Measures

#### 2.3.1. Initial Screening and Evaluation

Initial screening included a 20-minute telephone interview to determine participant eligibility. Subsequently, a multimeasure pretreatment evaluation was conducted, comprised of a semistructured sleep history interview to diagnose insomnia and the SCID-IV [20] to evaluate the presence of psychopathology. 

#### 2.3.2. Sleep Assessment

*Sleep Diaries*. Participants completed sleep diaries each morning upon rising throughout the experiment. From these diaries, total wake time (TWT; summation of time awake in bed including sleep-onset latency), total sleep time (TST), and SE were derived. Participants also monitored their sleepiness and alertness levels in the morning and evening using a “0” to “4” Likert scale as well as recording their saliva sample time.

*Polysomnography (PSG)*. Participants underwent a total of 10 nights of sleep laboratory assessment (see Section 2.2). The PSG montage included electroencephalographic (EEG; including C3, C4, O1, and O2), electromyographic (EMG; chin), and electro-oculographic (EOG; left and right: supraorbital ridge of one eye and the infraorbital ridge of the other) monitoring. Electrodes were referred to linked mastoids with a forehead ground, and interelectrode impedance was maintained below 5 kOhms. A Lifelines Trackit Recorders Mark 1 were used for data acquisition using Trackit software (hardware gain 500 +/− 2%; bandwidth 0.16–70 Hz) and PSG signals were digitized at a sampling rate of 256 Hz of 512 Hz using commercial software product (Harmonie, Stellate System, Montreal, Canada). Sleep recordings and limb movements were scored visually (Luna, Stellate System, Montreal, Canada) by qualified technicians using standardized criteria [26]. Recordings were made over 30-second epochs, and an independent scorer conducted reliability checks to insure a minimum of 85% interscorer agreement. Participants diagnosed with any other sleep disorder were excluded and referred to an appropriate sleep specialist. Respiration (airflow, tidal volume, and oxygen saturation) and anterior tibialis EMG readings were monitored during the first night of PSG recording in order to eliminate recordings made during sleep apnoea or periodic limb movements. Although sleep scoring was done before publication of the most recent guidelines [27], we chose to conserve the original scoring method, since it is more appropriate for research involving quantitative analyses of the EEG or finer techniques of EEG analyses (e.g., event-related potentials; ERPs).

Outcome measures (sleep-onset latency (SOL), wake after sleep onset (WASO), TST, and SE) were based on the average of baseline nights (BN1 and BN2: nights 1 and 2), first treatment nights (TR3 and TR4: nights 3 and 4), sleep stabilized nights (TR5 and TR6: nights 5 and 6), posttreatment nights (TR7 and TR8: nights 7 and 8), and follow-up nights (FUN9 and FUN10: nights 9 and 10).

*Power Spectral Analysis (PSA)*. PSA was conducted on EEG at C3 site only by computing fast Fourier transforms. EMG artefacts were detected automatically and rejected from the spectral analyses [28]. Further artefacts were eliminated by visual detection. Manual selection of periods of the night for PSA included all NREM and REM sleep as well as parts of each NREM sleep stage (1 to 4) of each sleep cycle (when available), excluding miniarousals (0.1–7 seconds), microarousals (7.1–14.9 seconds), arousals (15 seconds and longer), movement time, movements or artefacts, and the five minutes before and after a stage shift. Within a cycle, if no uninterrupted period of a specific sleep stage lasted longer than 10 minutes, a portion of this sleep stage was selected while excluding the first and last 40 seconds (two epochs) so not to include stage shifts in the analysis. 

A comparison between baseline and the introduction of treatment permits study of homeostatic processes occurring at the beginning of treatment. It is also possible that sleep recuperation occurs after the introduction of treatment. Clinical sleep data were derived from PSG night 2 (BN2), night 3 (TR3), and night 4 (TR4) only. PSA was computed for consecutive 4-second epochs, with a resolution of 0,25 Hz and an EEG segment length of 30 seconds. Data were cosine tapered, and fast Fourier transform windows were nonoverlapping. Frequencies were defined as follows: slow waves (0-1 Hz), delta (1–4 Hz), theta (4–7 Hz), alpha (7–11 Hz), sigma (11–14 Hz), beta-1 (14–20 Hz), beta-2 (20–35 Hz), gamma (35–60 Hz), omega (60–125 Hz), and total (0–125 Hz). Absolute power spectral values (*μ*V²) of REM and NREM sleep were log transformed to normalize the distributions.

*Actigraphy*. The actigraph is a watch-like device which records movement information over short periods by means of an accelerometer/microprocessor link. Presence of movement was interpreted as wake time and absence of movement as sleep time. The actigraphs used are from Cambridge Neurotechnology, AW-4. An algorithm (maximum sampling frequency 32 Hz, recording all movements over 0.05 g., filters set 3–11 Hz) enabled Sleepwatch software to estimate the sleep parameters using 1-minute epochs. 

#### 2.3.3. Cortisol Assessment

Salivary cortisol samples were drawn using a plastic tube. Each sample contained approximately 2 mL of saliva. Throughout the experimentation, samples were drawn 10 minutes before going to bed as well as 10 minutes after awakening. For home assessment, a kit of 14 plastic tubes was supplied weekly to each participant, who was instructed to collect salivary samples twice a day, not to eat or brush their teeth during the hour before collection, and to rinse their mouth with water 10 minutes before sampling. Then, they were instructed to put it in the appropriate plastic tube and to store it in their own refrigerator. Participants returned their 14 samples when they came to treatment sessions. In-lab and home salivary samples were stored at the Department of Biochemistry at the Glasgow Royal Infirmary and analyzed by an experienced biomedical technologist. When analyzed, samples were centrifuged (2500 rpm) for 10 minutes and the supernatant was frozen at −20c until assayed in the laboratory. These supernatants were radio immunoassayed using microencapsulated antibody and I-cortisol as a tracer. Cortisol level is expressed in nmol/mL.

#### 2.3.4. Insomnia Measure

Insomnia Serverity Index (ISI) [19] includes seven items. Ratings on a “0” to “4” point scale were obtained on the perceived severity of sleep-onset, sleep maintenance, and early morning awakening problems, satisfaction with current sleep patterns, interference with daily functioning, noticeable impairments attributed to sleep problems, and level of distress. The ISI score ranges from “0” to “28” with higher scores indicating more severe insomnia. This index has adequate psychometric properties and has been shown to be sensitive to changes in clinical trials of insomnia [29–31]. 

#### 2.3.5. Compliance Measures

Adherence to treatment protocol was evaluated with sleep diaries and actigraphy. A daily percentage of adherences to the prescribed time to go to bed as well as to arising time were computed separately for each participant and assessment device. Going to bed more than 15 minutes earlier and getting out of bed more than 15 minutes later than the prescribed sleep window was considered as nonadherence to the respective element of the sleep restriction procedure. A daily average in minutes of nonadherence time was also computed for each participant and assessment device.

#### 2.3.6. Treatment Response

Clinical judgments of treatment response were made according to the following criteria: (a) having a marked decreased in ISI score from baseline to posttreatment, (b) having sleep stabilized during treatment, and (c) presenting a significant increase in SE during treatment. Participants' responses were recorded as responder (meeting three of the above criteria), moderate responder (two criteria), or minimal responder (one criterion).

### 2.4. Data Analysis

Sleep diary data for four dependent variables (i.e., SOL, TWT, TST, and SE) were divided into consecutive series according to each period (i.e., baseline, treatment, and posttreatment) for each participant. An interrupted time series analysis (ITSA) [32] was conducted to statistically test whether the treatment was associated with a gradual (slope) or abrupt (level) change in the data series. Two comparisons of adjacent experimental periods were completed: (a) baseline versus treatment and (b) treatment versus posttreatment. To perform these analyses, ITSA models were developed using the AUTOREG procedure of SAS 9.1.3 [33], which uses a generalized least-squares regression method with residuals corrected for autocorrelation (serial dependency). Missing data were estimated within the model. Time, level, and slope effects were estimated following the recommendations of Huitema and McKean [34]. Autocorrelation of observations was studied for the first 12 lags. Final residuals were inspected to ensure that they were normally distributed and that they exhibited homogenous variance as well as no significant autocorrelation.

To study the physiological mechanisms of sleep restriction, statistical analyses were chosen as a function of (a) the objective, that is, to document the effect of sleep restriction on objective sleep, on subjective sleepiness and alertness, and on morning and evening cortisol levels, and (b) the nature of available data for each participant. For example, few data points are available for PSG, given the limited number of nights that each participant spent in the lab, while series of daily data are available from sleep diaries. 

Descriptive statistics were computed and visually inspected for PSG data for each two-night period spent in the laboratory except for the baseline nights where data were taken only for the second night because of a possible first night effect. For the PSA, statistical analyses were performed separately for participants who responded to treatment and for those who did not. Considering the small sample sizes, nonparametric statistics were used. The Friedman test evaluated potential statistical differences between the second baseline night (BN2) and the two first treatment nights (TR3 and TR4) for power spectral analysis variables of responders. In case of a significant Friedman test, post hoc analyses were performed using the Wilcoxon signed-rank test and a Bonferroni correction was applied, which resulted in a significance level of *P* < 0.017. The lack of data for some variables justified the direct use of the Wilcoxon test since the Friedman test could not be performed. In that case, the Bonferroni correction resulted in a significance level set at *P* < 0.025. This was the case for a few variables: NREM, REM, and stage 2 of the third cycle, stages 1 and 2 of the fourth cycle, and all variables for the minimal responder.

To study the longitudinal association between alertness, sleepiness, and sleep, Spearman correlations were calculated between subjective levels of alertness and sleepiness in the morning and the previous night's sleep variables (SOL, WASO, TWT, TST, and SE). Similar correlations were calculated between sleepiness at night and sleep variables. Finally, daily morning and evening cortisol levels were measured to ensure the reliability of these data, and z-scores were derived to facilitate comparisons between participants. Weekly means of standard scores were computed. Given the small sample size, no inferential analysis was performed. 

## 3. Results

### 3.1. Sleep Restriction Efficacy

Figures 1 and 2 show daily changes in SE and TWT for all participants throughout the experiment. Visual inspection of both SE and TWT over time shows extensive variability over nights in the sleep patterns of participants during baseline and no sleep improvement before treatment introduction. ITSA were performed on SOL, TWT, TST, and SE separately for each participant to determine if there was significant improvement after introducing treatment. The statistical modeling of these series explained an average of 79.6% of variance for SOL (*R* ^*2*^ range from 56.9% to 93.9%), 66.0% of variance for TWT (*R* ^*2*^ range from 47.7% to 90.0%), 50.4% of variance for TST (*R* ^*2*^ range from 24.8% to 77.5%), and 60.0% of variance for SE (*R* ^*2*^ range from 35.5% to 89.9%).

Results for the nature and direction of change for each sleep variable and participant are presented in Table 2. Four out of five participants presented a significantly decreased level of SOL from baseline to treatment (an average of 30 minutes). Moreover, all of them presented a significant decrease in TWT (an average of 96 minutes). Sleep efficiency increased significantly for three participants (an average of 15.9%) during treatment. Meanwhile, TST decreased significantly for three participants (an average of 56 minutes).

#### 3.1.1. Insomnia Severity

 Data presented in Table 3 indicated a decrease in severity from baseline to posttreatment for participants 1, 2, 3, and 5. Improvements were maintained at the 3-month follow-up although participant 2 was by then showing mild clinical insomnia. Participant 4 presented a severe insomnia at each assessment period.

#### 3.1.2. Course of Sleep Restriction

Sleep restriction was adapted as a function of individual response to treatment. Accordingly, the first sleep window length and its modification during treatment varied for each participant (see Table 3). Along with sleep window modifications, varied sleep restriction courses can be observed as sleep stabilization was attained: participants 1 and 2 presented the shortest time to stabilization and participant 3 took 16 days while the two other participants barely reached stabilization. 

#### 3.1.3. Compliance

Percentages of adherence varied greatly across individuals and weeks. Moreover, the adherence rate was lower when assessed using the actigraph than using the sleep diary. Deviations from the sleep window were different for each participant. The data show that four participants modified their sleep window during the week (see Table 3). According to actigraph measurements, participant 1 shortened his sleep window while participant 3 delayed his sleep window for an average of half an hour compared to the prescribed duration. Deviations of participant 4 indicated an increase in its sleep window length of about 90 minutes. Finally, participant 5 increased his sleep window in the morning.

#### 3.1.4. Treatment Response

Based on the clinical criteria described in the Data Analysis section, three participants responded to treatment (participants 1, 2, and 3), one had a minimal treatment response (participant 5), and one dropped out of treatment (participant 4). Therefore, participants 1, 2, and 3 were considered as being treatment responders and participants 4 and 5 as nonresponders.

### 3.2. Physiological Mechanisms of Sleep Restriction

#### 3.2.1. Objective Sleep

Means and standard deviations for PSG variables are presented in Table 4 for each assessment period. Objective data support ITSA of the sleep diary for most of the sleep variables in participants. Indeed, visual inspection of PSG data reveals a decrease in SOL and an increase in SE from the first nights of treatment spent in the laboratory to posttreatment. WASO seemed to improve similarly except at posttreatment. TST seemed to decrease during the first two nights of treatment but increased once sleep was considered stabilized and at posttreatment. For participants who had minimal treatment response, objective sleep measures indicate that both wake time and sleep time decreased from the very beginning of treatment. However, wake time remained superior to the clinical threshold of 30 minutes. 

Results for sleep stages indicate that the percentage of stage 2 sleep decreased slightly while the percentage of time asleep in stages 3 and 4 seemed to increase slightly between baseline and the first two nights of sleep restriction. REM sleep seemed to show the most marked increase at that time. These changes seemed to remain stable when sleep became stabilized. At posttreatment, percentage of time spent asleep in stage 2 appeared to return to baseline level while percentage of stages 3 and 4 decreased beneath the baseline level. For minimal responders, sleep stage changes were similar during the first night of in-lab treatment. Afterwards, stage 2 increased and stages 3-4 decreased drastically. Moreover, the proportion of REM sleep increased to 32% of the night for one participant.

Median power values for band frequencies from the second baseline night (BN2) and the two first treatment nights (TR3 and TR4) are presented in Table 5. PSA showed that, for cycles 1 and 2, all statistically significant Friedman test results were observed in the beta-1 and beta-2 band frequencies and appeared mainly during the first cycle. For beta-1, significant differences were observed for beta-1 in stages 2 (*χ* ^2^ (2) = 6.00, *P* = 0.05) and 3 (*χ* ^2^(2) = 6.00, *P* = 0.05) of the first cycle. Only beta-1 had a significant decrease for the second cycle across the three nights during NREM (*χ* ^2^(2) = 6.00, *P* = 0.05). Altogether, there seems to be an overall decrease across the three nights in beta-1 power during stages 2 and 3 of sleep of the first cycle and NREM sleep of the second cycle. For beta-2, significant differences were observed during NREM (*χ* ^2^(2) = 6.00, *P* = 0.05) and stage 4 (*χ* ^2^(2) = 6.00, *P* = 0.05) of the second stage. The medians seem to indicate a slight increase from BN2 to TR3 in beta-2 power followed by a decrease at TR4, which resulted in less power values at TR4 than at BN2 during stage 4 and NREM. However, post hoc analyses did not reveal any significant differences across nights and the *Z* value was similar in all comparisons. Due to the treatment procedure of restricting the time in bed, TR4 data were missing for cycles 3, 4, and 5 and TR3 data for cycle 5. Therefore, BN2 and TR3 data were compared using a Wilcoxon signed-rank test and on REM, NREM, and stage 2 only. No significant results emerged. 

Overall, a variable representing the mean power value of all the cycles was computed for each band frequency and sleep stage. As documented in Table 5, a few significant results were observed. During stage 2 of sleep, both beta bands (*χ* ^2^(2) = 6.00, *P* = 0.05) significantly differed across the three nights. A slight increase appears from BN2 to TR3 in beta-2 power followed by a decrease at TR4, which resulted in less power values at TR4 than at BN2. On the other hand, beta-1 power seems to decrease from BN2 to TR4. Beta-1 also significantly differed across nights during stage 3 (*χ* ^2^(2) = 6.00, *P* = 0.05) and NREM (*χ* ^2^(2) = 6,00, *P* = 0.05). Thus, beta-1 power seems to decrease across nights in stages 2, 3, and NREM among responders. Again, post hoc analyses revealed no significant results.

For minimal responders, BN2 data were missing for all the variables for the cycles from 1 to 5. Therefore, statistical analyses were performed only on TR3 and TR4. Results for the first 2 cycles and all cycles combined are shown in Table 5. In all analyses, no significant results could be observed. Again, because of the sleep restriction procedures, many data were missing from cycles 3 to 5.

#### 3.2.2. Perceived Sleepiness and Alertness in Relation with Sleep

Longitudinal associations between sleepiness and alertness in the morning, sleepiness at night, and sleep variables were assessed with correlational analyses.  Table 6 shows correlational coefficients between each variable for each participant during treatment. During baseline, there are only a few significant correlations, mainly between sleepiness at night and TWT and WASO (*P*s < 0.01). During treatment, participants 1, 2, and 3, who responded to treatment, presented several significant positive correlations between morning sleepiness and both SOL and TWT (*P*s < 0.0001 and 0.01, resp.) and TST and SE were negatively associated with sleepiness in the morning (*P*s < 0.01). These results show that greater sleepiness at night is associated with higher SE and shorter wake time during the night. Participant 2 presented significant associations as well between alertness in the morning and sleep variables (*P*s < 0.01, 0.05, and 0.001, resp.), showing that a high wake time was associated with a low level of alertness and a high sleep time was associated with a high level of alertness. Participant 3 had only a few significant associations. Participants 4 and 5, who did not respond well to treatment, had a few significant associations during treatment, mainly between sleepiness at night and WASO (*P* < 0.01) or TWT (*P* < 0.01). None of them presented significant associations with alertness in the morning.

#### 3.2.3. Morning and Evening Cortisol Levels

**Figure 3:**
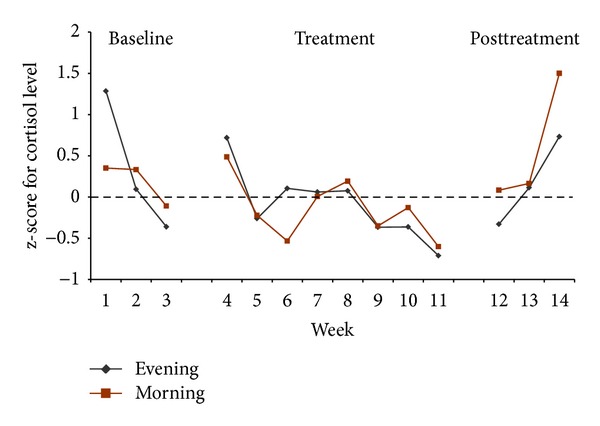
Evening and morning cortisol levels compared to their respective average for participants 1, 2, and 3. 0 as z-score means participant's average of cortisol levels. A negative z-score means a cortisol level lower than participant's average while a positive z-score means a cortisol level higher than participant's average of cortisol levels.

The mean morning cortisol level for the participants was 22.4 nmol/mL (SD = 11.4) and the evening level was 4.3 (SD = 5.1), which are within the normal range for these times of day. Overall, 69 of 262 evening saliva samples and 62 of 262 morning saliva samples were missing or unessayable, with saliva being contaminated before reaching the lab. Most of the missing saliva samples are from participant 4 who did not complete the experiment. Higher cortisol levels are associated with higher wake times.  Figure 3 showed the weekly mean morning and evening cortisol levels in standard scores for the three participants with an excellent response to sleep restriction. Visual inspection of these data suggests a tendency for both levels of cortisol to decrease before treatment. By the second week of treatment, both levels seemed lower than baseline as reflected by each participant's personal average which is represented by a standard score of 0. After treatment, both cortisol levels showed a tendency to increase. Participants 4 and 5 showed a similar trend in their cortisol levels in the morning. However, their cortisol levels in the evening tended to be higher, reaching an average of 0.35 by the end of treatment compared to a baseline level of −0.32. This trend is opposite to that seen in treatment responder.

## 4. Discussion

Our study illustrates that physiological mechanisms of sleep restriction therapy could be evaluated using an appropriate methodological strategy. By beginning measurements on the first night of treatment, it was revealed that sleep restriction might have a rapid impact on subjective sleep and on the physiological markers of sleep. First, the results showed that sleep restriction decreased total wake time sleep-onset latency and increased sleep efficiency. Second, these results showed that the subjective total sleep time is decreased by about one hour during the first week of treatment. Visual inspection of PSG data suggests that stage 2 decreases when introducing the treatment, while stage 3 seems to increase. An increase in REM sleep can also be observed. PSA indicated a change in beta-1 and -2 beginning with the second treatment night. Third, the results illustrated a potential action of sleep restriction on cortisol levels as both morning and evening cortisol levels seem to decrease during treatment. Fourth, with respect to sleepiness, the results show that greater sleepiness at night is associated with higher sleep efficiency and shorter wake time during the night. They also suggest that alertness in the morning is associated with previous sleep time. Finally, the results on sleepiness indicate that these associations were not present before treatment, suggesting that they are induced by sleep restriction. 

Objective sleep data obtained in the present study present similarities and divergences with results from other studies. They are similar to Cervena and colleagues [14] except that the increase in SWS in their study was clearer. PSA suggest that, when treatment is effective, beta-1 power decreases from baseline to the introduction of sleep restriction therapy. On the second night of treatment, both beta bands' powers seem to decrease. These PSA results converge with other studies [13, 14] that found a decrease after treatment in beta-1 and -2 while they diverge from the Krystal and Edinger study [9] that did not find a decrease in beta bands after CBT-I. Regarding powers of lower frequency bands (slow or delta), our study is more similar to Krystal and Edinger's findings [9] than to Cervena et al.'s finding [14]; the latter found an increase in SWS after treatment. Given that sleep restriction is used alone in our study while it is included in a multicomponent treatment in other studies, similarities between studies can be interpreted as being due to sleep restriction. Divergences might reflect an effect of another component of the CBT-I that was used in other studies. Nevertheless, our results support the assessment of beta-1 and -2 separately as it has been done in our previous studies [35, 36]. Cautiously, it could be suggested that this decrease in beta powers might also be an indicator of treatment efficacy or at least reflect a positive response to treatment.

Our results reporting an increase in REM sleep are similar to those of other studies reporting PSG data after treatment [14, 37]. However, our results make a further contribution by indicating that REM sleep begins to increase from the beginning of sleep restriction therapy and further increases during and after treatment. Based on the idea that REM sleep in insomnia is unstable and may contribute to sleep misperception [38], the increase in REM sleep observed during sleep restriction therapy could be interpreted as showing that sleep restriction consolidates REM sleep. Furthermore, one previous study found that REM sleep contributes to disrupt subjective perceptions of sleep and waking time [39]. Therefore, when the amount of REM sleep is increased and consolidated, it could contribute to the improvement of the subjective perception of sleep and wake time. However, the two participants who had a minimal response to sleep restriction treatment had an increase in REM sleep in the first nights. This suggests that other mechanisms are involved during treatment. The mechanisms underlying the increase in REM sleep seen during sleep restriction should be further investigated in larger studies.

Contrary to expectations, PSA do not indicate increase in SWS during sleep restriction therapy. This surprising result diverges from other studies [9, 14] that found an increase in SWS after CBT-I or that CBT-I led to a more rapid decline in delta power during NREM sleep. These two studies used a multicomponent treatment, while the methodology used in the present study isolated the effect of sleep restriction therapy. Therefore, one can argue that the SWS increase is not due to sleep restriction. Clearly, further studies will be needed to investigate the physiological mechanisms of action for other components of CBT-I. Nevertheless, because the sample of the present study is small, our results could also reflect a subsample of insomnia sufferers who happened to present an altered SWS.

The cortisol results seem congruent with an improvement in sleep during our sleep restriction treatment. Indeed, cortisol levels (evening and morning) are lower during treatment than at baseline for participants who had a treatment response. Moreover and most importantly, the decrease in cortisol levels can be detected very early in treatment. Cortisol levels are known to be higher for people with insomnia than for good sleepers [15, 16]. Thus, our results indicate that the use of sleep restriction alone, since it impacts cortisol levels, is a beneficial avenue for treating insomnia. Cortisol levels should thus be further evaluated during CBT-I.

In addition to the physiological sleep restriction therapy mechanisms, the findings highlight the rapid change observed in subjective sleep. Indeed, sleep restriction provides a rapid and marked decrease in wake time that is sustained during treatment. The findings also confirm a previously observed decrease in TST, quantifying that decrease at about an hour. Interestingly, these benefits in sleep were observed in spite of variations in the compliance data. It seems that individuals cope differently with difficulties encountered during treatment; some delayed their sleep window while some others shortened or changed the timing of the sleep window. Therefore, it appears that sleep restriction can be effective without a full application by the participant of the sleep restriction procedure.

Taken together, the data on PSG, sleepiness, and cortisol provide indications that sleep restriction decreases hyperarousal and cortical activity while increasing sleepiness to facilitate sleep. It is not clear, however, if these involve an increase of the homeostatic drive for all participants. The decrease in beta powers and in cortisol levels during treatment might reflect a decrease in hyperarousal. Contrary to both expectations and visual inspection of PSG, no increase in powers of lower frequency bands (slow or delta) indicative of greater homeostatic pressure was observed across nights. This, along with an increase in REM sleep, could reflect a malfunction of the homeostatic drive, implying that a decrease in wake time and sleep time will not generate the expected homeostatic sleep drive effect as previously suggested [8, 40]. Nevertheless, the sleepiness results suggest a relationship between sleep restriction and an increase in sleepiness at night, facilitating falling sleep and confirming a previous clinical report [41]. These data on perceived sleepiness are consistent with those of two studies [10, 12] as well as with the hypothesis that sleep restriction increases the homeostatic drive. Therefore, a strong and complete evaluation of the sleep restriction effect on homeostatic drive is warranted to more fully understand the sleep restriction mechanism.

These preliminary results possess some methodological limitations, although they are promising as a further step toward understanding sleep restriction mechanisms. A first limit concerns the small sample size that precludes obtaining strong statistical evidence of the mechanisms. Second, the procedure of daily assessing several variables during 10 to 12 weeks could have rendered the participants' tasks onerous, thus affecting data reliability. For this reason, sleep diary data were analyzed using ITSA. Third, cortisol data itself has several limitations: the trend for cortisol levels to decrease during baseline precludes definitive statements. The weekly adjustment of the sleep window might also have affected the evening cortisol level. However, although the time of going to bed differed for participants, the results followed a similar pattern. Moreover, both evening and morning cortisol levels were within the normal range for the time of day and the saliva methodology replicated that used in another study [34]: we see no reason to doubt the reliability of our cortisol results. Fourth, the fact that the first sleep restriction nights are spent in the laboratory might have influenced sleep data reported afterwards compared to other studies that did not use this strategy. On the other hand, this procedure has the advantage of standardizing the implementation of sleep restriction instructions by providing training for the procedure. 

## 5. Conclusion

This research evaluated the impact of sleep restriction on physiological markers of sleep, thus allowing a description of the putative physiological mechanisms for this treatment. The PSG and PSA results of the present study are innovative. They illustrate how a more in-depth investigation of the physiological variables related to sleep restriction could enlighten the knowledge on how sleep restriction works and on cortical activity in insomnia. The methodology used should be taken as a guideline for future studies. These findings illustrated the relevance of dismantling CBT-I to understand each component of treatment mechanism and enhance treatment efficacy. Future studies should investigate if the REM sleep increase observed with sleep restriction contributes to the improvement of sleep perception in insomnia. Circadian timing of sleep restriction and of other CBT-I components should also be further investigated in other studies. In addition, the sleepiness results, along with results obtained for TST, suggest that more attention should be given to the relationship between these two variables over the course of sleep restriction to evaluate a potential acute negative effect of sleep restriction. Finally, future studies should focus on empirically identifying sleep restriction mechanisms of action in order to increase efficacy and make relevant clinical recommendations concerning this promising form of therapy.

## Figures and Tables

**Table 1:** Summary content of the sleep restriction therapy.

Sleep restriction procedures	
(i) Sleep diaries used to estimate total sleep time (TST) and sleep efficiency (SE)	
(ii) Sleep window length = the average of the two last baseline weeks of TST	
(iii) The minimum sleep window duration is five hours	
(iv) Sleep window respected every night	
(v) Alarm clock used to ensure arising	
(vi) The sleep window	
(a) is increased for 15–20 minutes if SE ≥ 85%	
(b) is kept stable if SE is between 80% and 85%	
(c) is decreased to correspond to the total sleep time estimated if SE < 80%	
Session 1: sleep information and sleep restriction	
Aim: to transmit information about normal sleep, sleep disorders, and their effects and to begin sleep restriction therapy	
(i) Basic facts about sleep: sleep architecture, circadian rhythm and sleep homeostasis as regulators of sleep, and changes in sleep patterns over the life span	
(ii) Nature and causes of insomnia	
(iii) Introduction of sleep restriction therapy and determination of the first sleep window	
Session 2: sleep restriction	
Aim: to restructure sleep so that it meets individual needs and develops a stable pattern	
(i) Review previous week	
(ii) Continue sleep restriction	
(iii) Teach participants to modify their own sleep window	
(iv) Clarify the distinction between sleepiness and fatigue	
Session 3 and following ones until sleep stabilization: sleep restriction, developing natural sleep patterns	
Aim: same goal. In addition, teach participants to use sleep restriction	
(i) Continue sleep restriction	
(ii) Teach participants to modify their own sleep window	
(iii) Encourage fidelity to the new sleep schedule	
Last session: sleep restriction and therapeutic gain maintenance	
Aim: same goal. In addition, focus on further improvement and therapeutic gain maintenance	
(i) Continue sleep restriction	
(ii) Teach participants to modify their own sleep window	
(iii) Encourage fidelity to the new sleep schedule	
(iv) Review the concept of homeostatic pressure and more generally of the sleep restriction rationale	
(v) Maintain therapeutic gains and/or keep improving after treatment	

**Table 2:** Nature and direction of change between baseline, treatment, and post-treatment for each sleep variable and participant.

Sleep variables/participants	DFE	*R* ^2^	Treatment			Posttreatment		AR	AO
			Time	Level	Slope	Level	Slope		
Sleep-onset latency									
1	64	91.04	−0.56^ns^	−34.28***	0.30^ns^	7.27^ns^	1.52^ns^	1, 11	2
2	70	93.89	−0.65**	3.07^ns^	0.46^ns^	4.99^ns^	0.10^ns^	7	4
3	74	56.90	2.09^ns^	−69.77*	−2.72^ns^	37.23^ns^	−2.22^ns^	1	3
4	67	83.01	−0.18^ns^	−31.35***	0.38^ns^	−71.90**	33.30***	9	5
5	74	72.97	−0.28^ns^	−18.93*	0.34^ns^	−15.86^ns^	0.43^ns^	6, 10, 11	5

Mean	n/a	79.56	0.08	−30.25	−0.25	−7.65	6.63	n/a	n/a

Total wake time									
1	63	89.99	−0.71^ns^	−46.30***	0.52^ns^	21.22^ns^	−0.43^ns^	4	5
2	73	73.08	0.75^ns^	−155.94***	−0.38^ns^	8.22^ns^	−3.15^ns^	14	5
3	72	59.70	1.89^ns^	−87.79**	−1.94^ns^	43.12^ns^	−3.46^ns^	3, 9	4
4	72	59.34	−1.04^ns^	−140.16***	1.35^ns^	60.47^ns^	−6.89^ns^	8	0
5	79	47.73	−0.58^ns^	−50.56*	0.30^ns^	−37.65^ns^	5.17^ns^	9, 13	1

Mean	n/a	69.97	0.06	−96.15	−0.03	19.08	−1.75	n/a	n/a

Total sleep time									
1	62	77.54	0.77^ns^	−65.55**	0.48^ns^	−41.98^ns^	14.89*	14	5
2	71	61.44	−4.57^ns^	−0.31^ns^	6.19*	−109.06*	9.09^ns^	1, 10, 11	3
3	72	57.33	−0.44^ns^	−134.33***	2.41^ns^	−63.32^ns^	5.14^ns^	1	5
4	71	24.79	1.15^ns^	−60.08*	−1.43^ns^	−41.27^ns^	10.94^ns^	6, 12	0
5	79	30.95	0.43^ns^	−17.26^ns^	−0.39^ns^	35.12^ns^	5.41^ns^	5	2

Mean	n/a	50.41	−0.53	−55.51	1.45	−44.10	9.09	n/a	n/a

Sleep efficiency									
1	61	89.89	0.20^ns^	7.10**	−0.13^ns^	−3.83^ns^	0.18^ns^	0	5
2	71	71.57	−0.28^ns^	36.08***	0.13^ns^	−6.46^ns^	1.03^ns^	1, 10	4
3	72	50.96	−0.30^ns^	10.49^ns^	0.37^ns^	−9.83^ns^	0.80^ns^	1, 9	4
4	68	51.79	0.06^ns^	18.60**	−0.10^ns^	−20.02^ns^	2.55^ns^	12, 14	3
5	79	35.47	0.12^ns^	7.19^ns^	−0.05^ns^	8.02^ns^	−0.61^ns^	1, 9, 13	0

Mean	n/a	59.94	−0.04	15.89	0.04	−6.42	0.79	n/a	n/a

DFE: degree of freedom; AR: autocorrelation; AO: number of outliers; ns: not significant.

*P < 0.05; **P < 0.01; ***P < 0.001.

**Table 3:** Descriptive information of participants and treatment course.

				ISI			Sleep window		^ ^	Respect to time off bed (min)		Respect to arising time (min)	
		Age	Insomnia duration						Sleep stabilisation (days)	Actigraph M (SD)	Sleep diary M (SD)	Actigraph M (SD)	Sleep diary M (SD)
				B	Post	Fu3	Duration of the 1st	Modification	^ ^				
		(Years)					^ ^	^ ^	^ ^			
P1		22	17	11	6	7	6:30	Weekly ↑ by 15 min	6	16.4 (16.7)	18.3 (34.5)	−8.9 (17.7)	0.9 (28.4)
P2		36	6	18	10	10	5:00	Weekly ↑ by 15 min until week 6	9	4.4 (11.4)	−0.6 (14.1)	7.7 (11.1)	13.6 (15.7)
P3		62	5	17	13	8	6:40	↑ by 15 min at weeks 3, 5, and 6	16	24.1 (24.2)	−1.6 (2.7)	23.8 (19.8)	13.4 (11.8)
P4		36	15	15	0	0	6:00	↑ by 15 min at weeks 3 and 4	n/a	−21.2 (0.0)	−9.5 (19.2)	72.2 (0.0)	5.38 (18.6)
P5		53	20	11	6	7	6:15	Weekly ↓ by 15 min for 3 weeks; then ↑ by 15 min	25	−1.98 (17.9)	13.5 (11.8)	46.0 (25.0)	29.0 (15.8)

ISI: Insomnia Severity Index; B: baseline; Post: posttreatment; Fu3: 3-month follow-up; ↑: increase; ↓: decrease; min: minute; Respect to time off bed: a score of “0” means a perfect respect of the time off bed. A positive score means going to bed later than the prescribed time while a negative score means going to bed earlier; Respect to arising time: a score of “0” means a perfect respect of the prescribed time to get out bed in the morning. A positive score means getting out of bed later than prescribed time in the morning while a negative score means getting out of bed earlier than prescribed.

**Table 4:** Means (M) and standard deviations (SD) of polysomnographic data.

Sleep variables	Evaluation periods				
	Baseline (BN2) M (SD)	Treatment		Posttreatment M (SD)	Follow-up M (SD)
		1st nights (TR3, TR4) M (SD)	Sleep stabilized M (SD)		
Treatment responders (*n* = 3, except at follow-up *n* = 2)					
SOL (min)	51.8 (69.9)	13.0 (15.5)	7.7 (7.6)	14.8 (11.1)	46.0 (65.0)
WASO (min)	55.1 (25.9)	26.2 (14.3)	17.5 (17.5)	42.0 (56.9)	32.5 (29.9)
TST (min)	330.1 (80.5)	306.7 (32.6)	348.5 (18.6)	350.9 (37.3)	371.5 (29.4)
SE (%)	74.8 (16.3)	88.2 (4.4)	92.5 (4.6)	86.3 (11.6)	85.2 (14.5)
% stage 2	52.1 (6.4)	49.9 (6.9)	46.5 (4.9)	55.3 (6.8)	54.7 (8.1)
% stages 3-4	21.9 (5.1)	23.7 (7.5)	24.6 (3.9)	14.0 (19.8)	15.6 (8.1)
% REM	17.8 (5.0)	22.7 (5.5)	25.2 (2.8)	27.5 (4.2)	26.1 (2.4)

Nonresponders (*n* = 2 for baseline and 1st nights, then, *n* = 1)					
SOL (min)	17.1 (13.0)	18.0 (7.7)	6.3 (6.7)	5.3 (0.4)	28.0 (24.5)
WASO (min)	115.5 (73.6)	26.3 (8.5)	64.3 (27.9)	55.8 (2.5)	64.8 (15.2)
TST (min)	302.4 (85.1)	289.1 (39.2)	285.0 (25.5)	312.8 (1.1)	385.8 (40.7)
SE (%)	68.8 (14.9)	86 (3.5)	79.5 (6.4)	83.0 (0.0)	80.0 (8.5)
% stage 2	56.0 (5.3)	54.8 (8.7)	70.1 (1.6)	56.0 (4.9)	63.0 (1.8)
% stages 3-4	15.8 (4.4)	18.7 (10.2)	2.0 (1.7)	10.5 (3.6)	6.2 (5.1)
% REM	21.9 (8.0)	21.2 (5.3)	24.9 (0.3)	32.2 (2.0)	27.7 (6.9)

BN2: baseline night 2; TR3: the third night in lab and the first of treatment; TR4: the fourth night in lab and the second of treatment; SOL: sleep-onset latency; WASO: wake time after sleep-onset; TST: total sleep time; SE: sleep efficiency; REM: rapid eye movement; min: minutes.

**Table 5:** Median (range) power values for responders for beta-1 and beta-2 band frequencies.

		BN2		TR3		TR4	
	Sleep stages	Median	Range	Median	Range	Median	Range
Beta-1							
Cycle 1	NRem	2.41	1.62–12.14	2.09	1.92–9.06	1.48	1.13–2.98
	Rem	1.83	0.78–5.93	1.88	0.89–6.37	2.45	0.58–3.11
	1	3.01	1.06–7.54	2.93	2.93-2.93	1.80	1.14–2.45
	2*	4.27	2.99–13.88	3.29	2.22–11.89	3.15	1.31–5.13
	3*	2.30	2.10–9.09	1.91	1.59–6.97	1.34	1.29–3.95
	4	1.53	1.40–7.45	1.62	1.48–5.09	3.02	2.90–4.64
Cycle 2	NRem*	3.86	1.72–11.04	3.44	1.33–4.75	1.81	1.14–2.55
	Rem	1.65	0.73–5.28	1.11	0.98–6.34	1.33	0.66–2.86
	1	1.40	1.40-1.40	1.55	1.55-1.55	1.88	1.40–2.36
	2	8.45	2.31–14.80	3.44	2.31–6.78	2.79	1.67–5.13
	3	2.02	1.42–8.16	5.10	1.15–9.04	1.75	1.28–3.83
	4	3.37	1.27–5.47	2.44	0.95–3.92	1.44	0.76–1.56
All cycles	NRem*	3.21	1.76–11.99	2.77	1.69–7.43	2.38	1.19–4.22
	Rem	1.57	0.77–5.78	1.52	1.02–6.36	1.67	0.61–3.04
	1	3.46	1.14–7.05	2.71	1.33–7.25	2.39	0.95–3.19
	2*	3.53	2.23–14.64	3.30	2.18–10.10	3.19	1.51–5.77
	3*	2.09	1.51–8.47	1.91	1.34–6.79	1.57	1.33–3.87
	4	1.53	1.37–5.96	1.62	1.22–4.14	1.38	0.92–1.62

Beta-2							
Cycle 1	NRem	0.75	0.56–3.27	1.01	0.92–4.08	0.51	0.51–1.10
	Rem	1.13	0.86–4.01	1.04	0.89–5.29	0.98	0.60–1.64
	1	1.43	0.87–5.64	1.08	1.08-1.08	1.16	0.93–1.38
	2	1.25	0.89–3.13	1.39	1.31–5.82	1.19	0.66–2.09
	3	0.67	0.63–2.25	0.80	0.58–2.46	0.48	0.47–1.15
	4	0.56	0.50–2.17	0.84	0.59–2.48	1.18	0.96–1.61
Cycle 2	NRem*	1.78	0.67–2.51	1.00	0.47–1.89	0.67	0.42–0.93
	Rem	1.11	0.79–3.24	0.95	0.84–5.70	0.86	0.53–1.55
	1	1.43	1.43-1.43	1.35	1.35-1.35	1.00	0.92–1.07
	2	3.01	0.75–4.51	1.00	0.78–5.43	0.70	0.55–1.41
	3	0.68	0.53–2.18	1.49	0.48–2.50	0.60	0.45–1.14
	4*	1.09	0.47–1.71	1.04	0.49–1.59	0.53	0.32–0.75
All cycles	NRem	1.14	0.77–3.01	0.96	0.90–2.76	0.75	0.52–1.36
	Rem	1.11	0.82–3.59	1.00	0.88–5.72	0.92	0.58–1.80
	1	1.39	1.25–5.09	1.27	1.15–9.01	1.06	1.03–2.78
	2*	1.10	0.75–3.07	1.29	1.18–3.73	0.85	0.58–1.64
	3	0.66	0.54–2.20	0.80	0.52–2.35	0.55	0.47–1.14
	4	0.56	0.49–1.82	0.84	0.54–1.75	0.51	0.35–0.77

BN2: baseline night 2; TR3: the third night in lab and the first of treatment; TR4: the fourth night in lab and the second of treatment; NREM: nonrapid eye movement; REM: rapid eye movement. *Significant results at *P* ≤ 0.05 between nights for the sleep stages targeted.

**Table 6:** Correlation coefficients between subjective sleepiness, alertness, and sleep variables for each participant during treatment.

Participants/alertness and sleepiness	Sleep variables				
	SOL	WASO	TWT	TST	SE
Participant 1					
Alertness	−0.30*	0.13	−0.07	0.19	0.12
Sleepiness	0.51***	0.01	0.35**	−0.37**	−0.42
Sleepy at night	−0.39**	−0.29*	−0.39**	−0.02	0.32
Participant 2					
Alertness	−0.41**	−0.28*	−0.32*	0.53***	0.35**
Sleepiness	−0.01	0.26	0.28*	−0.17	−0.28*
Sleepy at night	−0.29*	−0.07	−0.07	−0.07	0.01
Participant 3					
Alertness	−0.02	−0.28	−0.22	0.25	0.21
Sleepiness	−0.02	0.32*	−0.02	−0.16	0.01
Sleepy at night	−0.16	0.31*	0.05	−0.08	−0.04
Participant 4					
Alertness	0.01	−0.27	0.08	0.29	0.03
Sleepiness	0.13	0.55***	0.17	−0.52***	−0.28
Sleepy at night	−0.30	−0.16	−0.05	0.18	0.09
Participant 5					
Alertness	−0.13	−0.44**	−0.28	0.29	0.31*
Sleepiness	−0.07	0.39**	0.17	−0.17	−0.19
Sleepy at night	−0.25	−0.27	−0.38**	0.24	0.36*

SOL: sleep-onset latency; WASO: wake after sleep-onset; TWT: total wake time including SOL, WASO, and early morning awakening; TST: total sleep time.

*P < 0.05; **P < 0.01; ***P < 0.001.
